# Simulation of dB/dt‐Over‐Electric Field Cardiac Magnetostimulation Safety Ratios in 75 Body Models and 18 Gradient Systems

**DOI:** 10.1002/mrm.70130

**Published:** 2025-10-07

**Authors:** Valerie Klein, Jonathan Edmonson, Mathias Davids, Natalie G. Ferris, Matthias Gebhardt, Dominik Rattenbacher, Johan S. van den Brink, Michael Steckner, Lawrence L. Wald, Bastien Guérin

**Affiliations:** ^1^ A. A. Martinos Center for Biomedical Imaging, Department of Radiology Massachusetts General Hospital Charlestown Massachusetts USA; ^2^ Harvard Medical School Boston Massachusetts USA; ^3^ Medtronic Cardiac Rhythm Management Minneapolis Minnesota USA; ^4^ Harvard‐MIT Division of Health Sciences and Technology Cambridge Massachusetts USA; ^5^ Siemens Healthineers Erlangen Germany; ^6^ Philips Healthcare Best the Netherlands; ^7^ MKS Consulting Beachwood Ohio USA

## Abstract

**Purpose:**

The IEC 60601‐2‐33 standard provides consensus‐based safety provisions for MRI equipment. Protection of patients against cardiac stimulation (CS) is based on limiting the maximum E‐field induced by MRI gradient coils. In practice, this is achieved by imposing a conservative dB/dt threshold on any gradient waveform. The dB/dt‐over‐E‐field conversion ratio currently used in IEC 60601‐2‐33 was derived in a homogeneous ellipsoid exposed to a uniform B‐field and is 10 (T/s)*(V/m)^−1^. This limit is becoming increasingly restrictive in high performance clinical systems. We therefore evaluate dB/dt‐over‐E‐field ratios in realistic body models and coils using state‐of‐the‐art electromagnetic simulations.

**Methods:**

We performed two independent simulation studies in a total of 75 realistic body models and 13 commercial gradient systems and derived dB/dt‐over‐E‐field ratios in the heart. We thresholded the E‐field maps to mitigate the impact of staircasing artifacts in boundary voxels between the myocardium and the lungs.

**Results:**

Thresholding the E‐field maps at the 99th percentile E‐field value (E99) eliminates staircasing artifacts in both simulation studies. Study #1 predicts a larger range of dB/dt‐over‐E99 ratios (13–53 (T/s)*(V/m)^−1^) than study #2 (12–35 (T/s)*(V/m)^−1^). Despite differences in EM solvers, body models, coils, mesh resolution, and post‐processing, both studies find similar worst‐case ratios of dB/dt‐over‐E99 of 12–13 (T/s)*(V/m)^−1^.

**Conclusion:**

Our simulations of dB/dt‐over‐E‐field ratios for cardiac safety in MRI cover a large range of realistic clinical scenarios. An increase of the allowable dB/dt beyond the current CS limit in IEC 60601‐2‐33 may be feasible.

## 1 Introduction

Magnetic resonance imaging uses magnetic gradient fields switched in the low kHz frequency range (dB/dt) for signal encoding and spin preparation. This switching of magnetic fields induces electric fields (E‐fields) in the patient that can stimulate excitable tissues, including the heart (cardiac magnetostimulation, CS) [1, 2, 3, 4, 5]. Safety protection of patients against CS is implemented on scanners in the form of dB/dt limits that are enforced both during sequence preparation and scanning. Those limits can significantly reduce the effective gradient performance expressed as the gradient strength (G) achievable at a given slew rate (S) compared to the hardware capability (G_max_, S_max_). dB/dt protection against peripheral nerve stimulation (PNS) has historically been the most limiting restriction, however, the dB/dt CS limit imposed by IEC 60601‐2‐33 [6] has been found to be increasingly restrictive for systems operating at or above G_max_ = 80 mT/m and S_max_ = 200 T/m/s. For example, the IEC CS limit is reached before the PNS limit by the Connectome scanner (G_max_ = 300mT/m, S_max_ = 200 T/m/s) for rise times greater than 1.5 ms [7].

Results by our group and others suggest that the current IEC CS limit may be overly conservative [8, 9, 10, 11]. For example, Irnich estimated that cardiac tissue has a greater E‐field rheobase E_rheo_ (i.e., the myocardial E‐field threshold at long stimulus durations) of 60 V/m [10] than peripheral nerves (E_rheo_ = 6.2 V/m for large myelinated nerves [12]). Such a rheobase difference should result in substantially higher CS thresholds than PNS thresholds, especially for short stimuli. In line with this, recent measurements and simulations in pigs by our group found CS thresholds ~11‐fold greater than the IEC dB/dt CS limit at an effective stimulus duration of 0.45 ms [9, 11].

Unlike PNS [4, 13], CS cannot be safely measured in human volunteers. Instead, CS limits rely exclusively on a combination of electromagnetic (EM) field modeling and animal experiments. In the early 1990s, Reilly compiled various CS experiments in animals using electrodes to stimulate the heart (different species, electrode locations, stimulation waveforms and experimental setups) [2]. He analyzed this body of experimental data and concluded that the E‐field rheobase for the most sensitive percentile of the human population was the same as the theoretical rheobase of a 20‐μm myelinated peripheral nerve fiber, that is, E_rheo_ = 6.2 V/m [2]. The IEC included a safety factor of 3X in this value, resulting in an IEC 60601‐2‐33 limit for the maximum acceptable myocardial E‐field rheobase of E_rheo_ = 2 V/m [6]. Using the assumption that CS thresholds are log‐normally distributed across the population [2], it was postulated that following this limit should lead to a probability of ectopic heartbeat induction of < 10 ppb. In IEC 60601‐2‐33, the CS E‐field limit for arbitrary waveforms characterized by an effective stimulus duration *t*_*s,eff*_ is given by:

(1) $$E<\frac{2\;V/m}{1-\exp\left(-\frac{\tau_{s,\mathrm{eff}}}{3\;\mathrm{ms}}\right)},$$

where *t*_*s,eff*_ is the ratio between the peak‐to‐peak gradient field variation and the maximum field derivative during a single gradient waveform period [6] (for a bipolar trapezoidal waveform, this corresponds to twice the gradient rise time).

To convert the E‐field limit of Equation (1) into the more practical dB/dt metric, Reilly performed analytical EM field calculations in a homogeneous ellipsoid representing “the torso of a large man” [2, 3]. He calculated E‐fields at the heart location within the ellipsoid induced by B‐fields that were uniform across the ellipsoid's cross‐section and found that the maximum E‐field induced at the heart location by a B‐field switched at dB/dt = 100 T/s was 12.2 V/m [2, 3]. From these calculations, the dB/dt‐over‐E‐field ratio, that is, the conversion factor between the maximum E‐field in the heart and the dB/dt metric, was approximated at 10 (T/s)*(V/m)^−1^. Combined with Equation (1), this results in the following IEC dB/dt limit for CS:

(2) $$\frac{\mathrm{dB}}{\mathrm{dt}}<\frac{20\;T/s}{1-\exp\left(-\frac{\tau_{s,\mathrm{eff}}}{3\;\mathrm{ms}}\right)}.$$

The IEC dB/dt limit is evaluated on a cylinder with a 20 cm radius placed along the patient long axis (the IEC “compliance volume” [6]).

The EM calculations performed by Reilly in the 1990s to estimate the dB/dt‐over‐E‐field ratio [2, 3] were helpful to establish practical safety limits, which, at the time, were well above the achievable gradient performance. However, Reilly modeled neither realistic gradient fields nor realistic heterogeneous body models. Since the performance of clinical systems keeps improving and the gap between the IEC CS limit and the achievable gradient performance decreases, we propose to revisit those simplifications using state‐of‐the‐art modeling methods. In this work, we calculate dB/dt‐over‐E‐field MRI CS safety ratios in 75 detailed body models with varying body shape, size, weight, age, and ethnicity, and for 18 realistic whole‐body gradient systems.

## 2 Methods

We performed two independent simulation studies with different EM solvers, body models, and gradient systems to derive a consensus proposal for an updated IEC dB/dt‐over‐E‐field CS safety ratio. VK ran study #1 and JE ran study #2 in an independent manner.

### 2.1 Body Models

**FIGURE 1:**
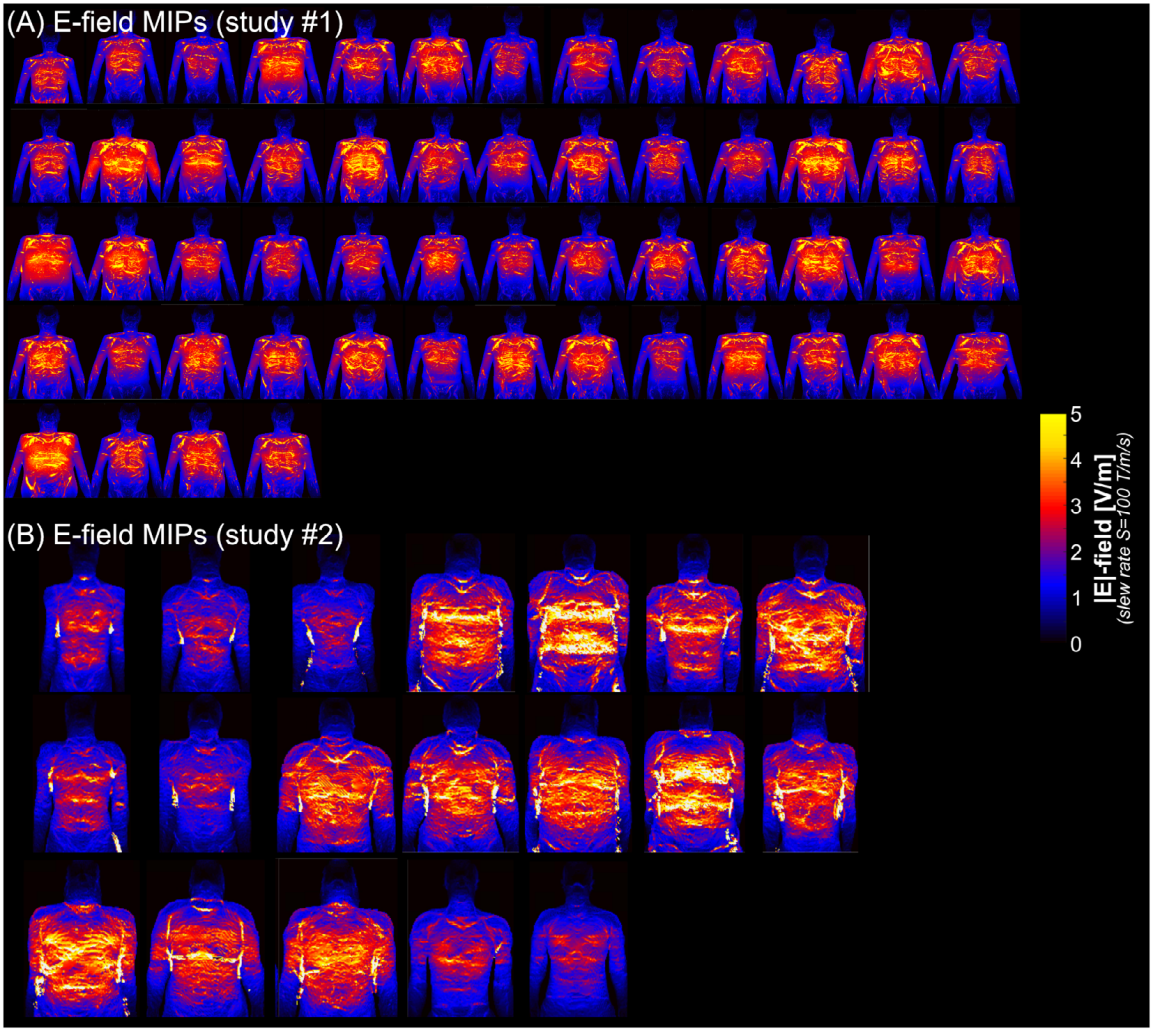
(A) E‐field maximum intensity projections (MIPs, coronal view) in the 56 XCAT body models simulated in study #1 for the X‐gradient of GC10 at a slew rate of 100 T/m/s (head landmark). (B) E‐field MIPs simulated in study #2 (19 body models, X‐gradient of GC15) at a slew rate of 100 T/m/s (head landmark). All E‐fields are scaled to the same color map.

In simulation study #1, we used 56 XCAT body models [14] (33 male, 23 female, 18–78 years, BMI 18–39, weight 52–120 kg, height 153–190 cm, Asian (*N* = 1)/Black (*N* = 8)/White (*N* = 25)/unspecified (*N* = 22) ethnicities represented) (Figure 1). Those models contain ~90 tissue classes assigned with electrical conductivity values from the IT'IS low‐frequency database [15] and were voxelized with an adaptive, non‐uniform mesh size of 1–8 mm (1 mm isotropic in the heart). Simulation study #2 used the Ella and Duke Virtual Family Project models, the male and female Visible Human Project (VHP) models as well as 15 volumetric morphs of the male and female VHP models. This resulted in 19 models in total spanning the 2nd to 97th percentiles of the adult population in height (154–187 cm) and weight (42–114 kg). The default heart volumes of each of these 15 volumetric morph VHP models were replaced with those from MRI scans of heart failure patients who have bigger hearts, which leads to more conservative E‐field estimates. Models comprise > 20 tissue classes with electrical parameters also assigned using the IT'IS low‐frequency material database [15] and were voxelized at 5 mm isotropic resolution. Overall, the body models simulated in studies #1 and #2 cover a realistic range of heights and weights, which can be seen by comparison with the CAESAR database [16] comprising data from 5000 North American and European adults (Figure S1A–C).

### 2.2 Gradient Coils (GC)

In study #1, we simulated EM fields for single axes (X, Y, and Z axes simulated individually) as well as the X + Y + Z combination of 13 actively shielded commercial whole‐body gradient systems (Siemens Healthineers, Erlangen, Germany, and Philips, Best, Netherlands), referred to as GC1‐GC13 (inner diameters: 64–92 cm, lengths: 106–160 cm). Study #2 simulated five symmetric idealized shielded GC sets (single axes and the *X* + *Y* + *Z* axis combination) with coil lengths of 110 cm, 150 cm, 190 cm and inner coil diameters of 66–70 cm (60 cm bore diameter) and coil lengths of 110 cm and 150 cm with inner coil diameters of 76–80 cm (70 cm bore diameter). We refer to these gradient systems as GC14‐GC18. The GCs simulated in studies #1 and #2 cover a realistic range of diameters and lengths (Figure S1D).

### 2.3 EM Field Solver

We solved E‐fields in study #1 using MFEM [17] for a coil current of 1 kHz frequency (sinusoidal waveform) and current amplitude corresponding to a gradient slew rate of 100 T/m/s. We placed the body models in head‐first supine position in the GCs and simulated 41 landmark locations for all body model/GC combinations with 5‐cm increments along the spatial z‐direction to determine the worst‐case landmarks corresponding to the highest E‐field in the myocardium. B‐fields were computed using Biot‐Savart's law in the IEC compliance volume [6] (20‐cm radius cylinder). Peak dB/dt values were computed by scaling the peak |B|‐field magnitude by (100 T/m/s)/G_eff_, where G_eff_ is the gradient efficiency of the coil at isocenter. In study #2, a custom Matlab code was used to compute the magnetic vector potential which was then fed into the SEMCAD X V14.8.6.1 low‐frequency magneto quasi‐static solver to calculate the E‐field. Simulations were performed for head‐first supine body positions at 9 landmark locations per body model/GC combination (10‐cm increments along the z‐direction). Peak dB/dt values were computed as for study #1.

### 2.4 Calculation of Probability of CS Adverse Event

IEC 60601‐2‐33 assumes that CS thresholds in units of E‐field rheobase are log‐normally distributed across the population [6]. Reilly suggested a median value of 12 V/m, and 6 V/m for the 1st percentile of this distribution [2]. In a first step, we use Reilly's E‐field threshold distribution to estimate the distribution of CS thresholds in units of dB/dt, a quantity that is easy to measure and control in MRI. To this end, we convolve Reilly's E‐field threshold distribution with our estimated distribution of dB/dt‐over‐E‐field ratios, resulting in a distribution of dB/dt threshold values. This distribution allows us to assess the probability that a given dB/dt value is supra‐threshold, that is, the probability that this dB/dt value causes a cardiac adverse event. Equivalently, we can compute the adverse event probability for different choices of the dB/dt‐over‐E‐field ratio, assuming an E‐field rheobase of 2 V/m (as in IEC 60601‐2‐33 [6]). For example, a dB/dt‐over‐E‐field ratio of 10 (T/s)*(V/m)^−1^ yields an effective dB/dt limit of 20 T/s. The probability of a CS adverse event for this ratio is thus simply the area (integral) of the distribution of dB/dt threshold values up to 20 T/s.

## 3 Results

**FIGURE 2:**
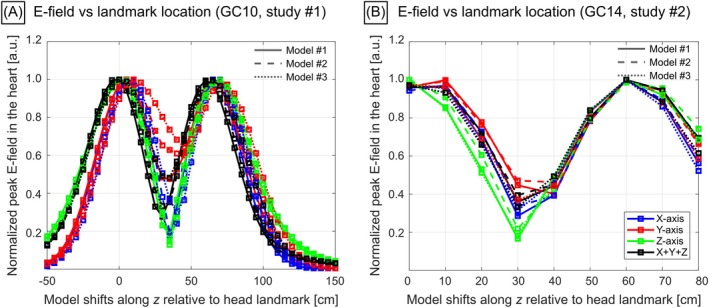
Normalized E‐field values induced in the myocardium of three body models (different line styles) of study #1 by GC10 (A) and three models of study #2 by GC14 (B) at different positions of the body model relative to the head landmark. The E‐field in the myocardium reaches peak values when the heart is positioned in locations of peak gradient B‐field magnitude (for X‐ and Y‐gradient coils, this corresponds to the location of the gradient coil's “eye”).

As expected, the induced E‐field maps are highly heterogeneous in the body, strongly shaped by the local tissue distributions, and are greater in large subjects as shown in the MIP images (Figure 1). Figure 2 shows E‐fields induced by GC10 and GC14 in the myocardium of one body model of study #1 and study #2 at different landmark locations. In both studies #1 and #2, peak myocardial E‐fields were reached by placing the heart roughly below one of the gradient coil's “eyes” (for *X*‐ and *Y*‐axis coils) or below regions of highest winding density (*Z*‐axis coils).

**FIGURE 3:**
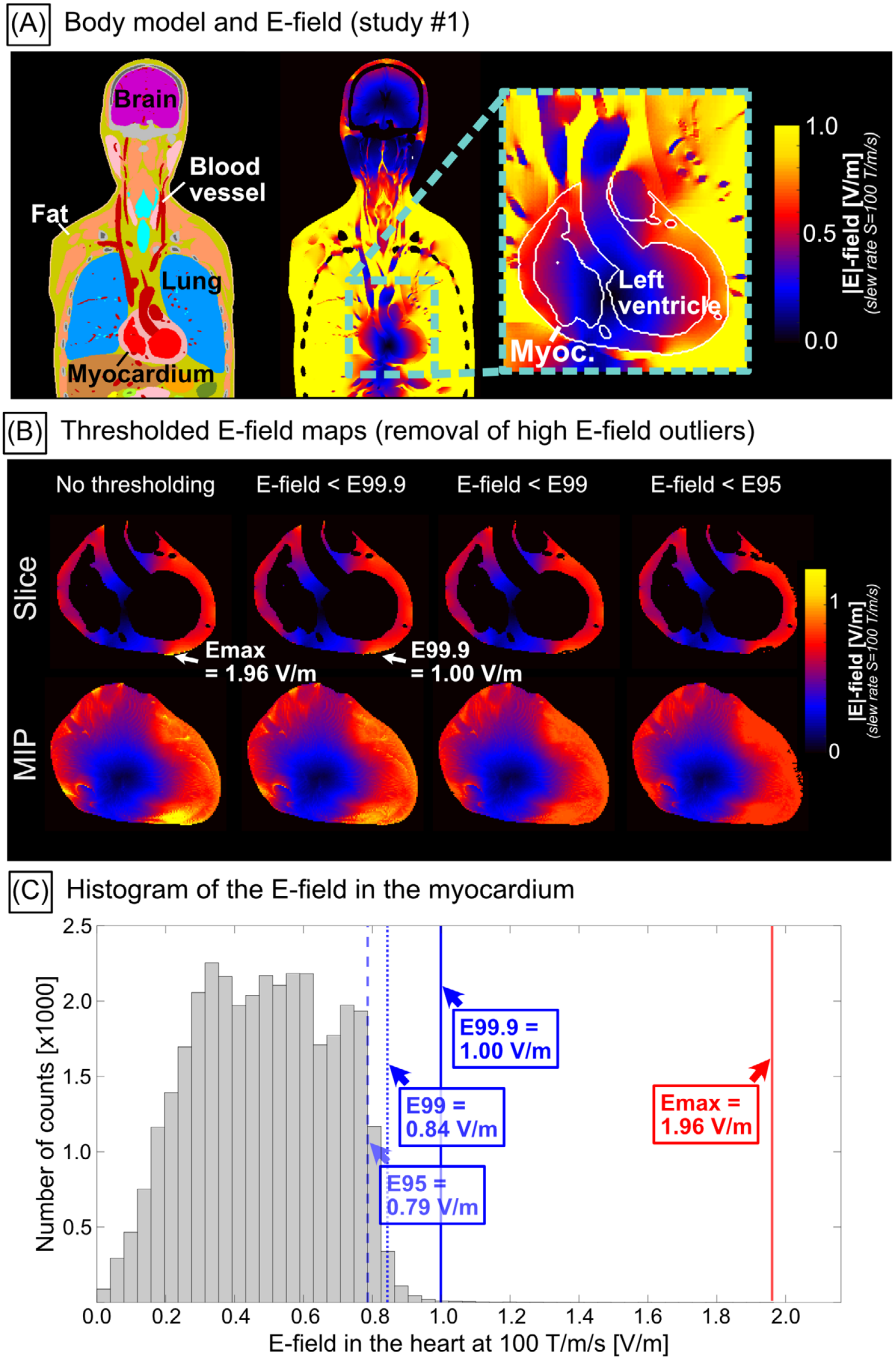
(A) Coronal slices of XCAT body model #157 and the E‐field induced by the Y‐gradient of GC4 at 100 T/m/s (study #1). (B) E‐field maximum intensity projections (MIPs, top row) and slices (bottom) in the myocardium thresholded at different E‐field percentile values. Staircasing artifacts can cause high E‐field values at tissue boundaries. Thresholding of the E‐field maps by percentile values reduces these E‐field outliers. (C) Distribution of E‐field values across all voxels of the myocardial volume. The histogram has a long tail toward high E‐field values, with a high single‐voxel maximum Emax caused by staircasing.

**FIGURE 4:**
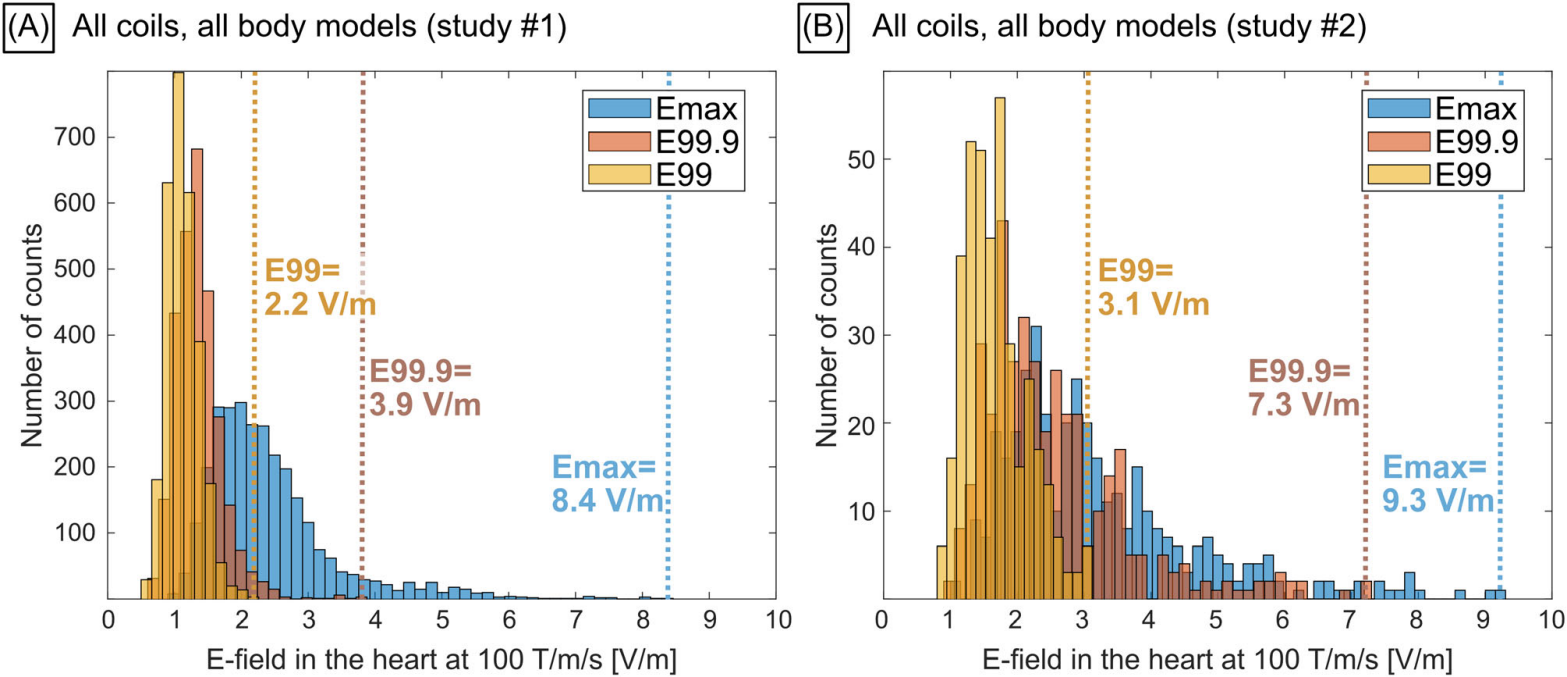
(A) Distribution of maximum E‐field (Emax), the 99.9th (E99.9) and 99th percentiles (E99) in the myocardium for all 56 body models and 13 gradient systems (X, *Y*, *Z*, *X* + *Y* + *Z* axis combinations) modeled in study #1. (B) Same plot for study #2. Vertical lines point to the peak values of each E‐field distribution. All E‐fields were scaled to match a gradient slew rate of 100 T/m/s per gradient axis, or a total slew rate of 100 T/m/s for the *X* + *Y* + *Z* axis combination. The Emax histograms have long tails to the right, meaning that some body model/gradient coil combinations have substantially higher peak E‐field values than most other combinations. Those Emax outliers are likely caused by staircasing.

Figure 3A shows coronal slices of one of the XCAT voxel models (study #1, GC4). The heart is located almost at body center, resulting in significant shielding by the surrounding body tissues. Artificially high E‐field values can occur in tissue boundary voxels, for example between the myocardium and the lungs and between the myocardium and the blood pool. Those values result from staircasing artifacts unavoidable in finite element simulations and are outliers to be removed (Figure 3B,C). Masking the myocardial E‐field maps above the 99th percentile threshold resolves this problem without removing too much data, as shown in Figures 3B and S2. E99 thresholding removes 1% of the voxels in the myocardium. A typical heart contains ~300 000 1‐mm isotropic voxels, thus E99 thresholding removes ~3000 voxels. Figure 4 provides another way to look at the data: Both the peak E‐field Emax (Emax = E100) and the 99.9th percentile E‐field E99.9 histograms have long tails, that is, some body model/GC combinations exhibit high Emax and E99.9 values compared to the mean, whereas the E99 histogram removes those high‐value outliers.

**FIGURE 5:**
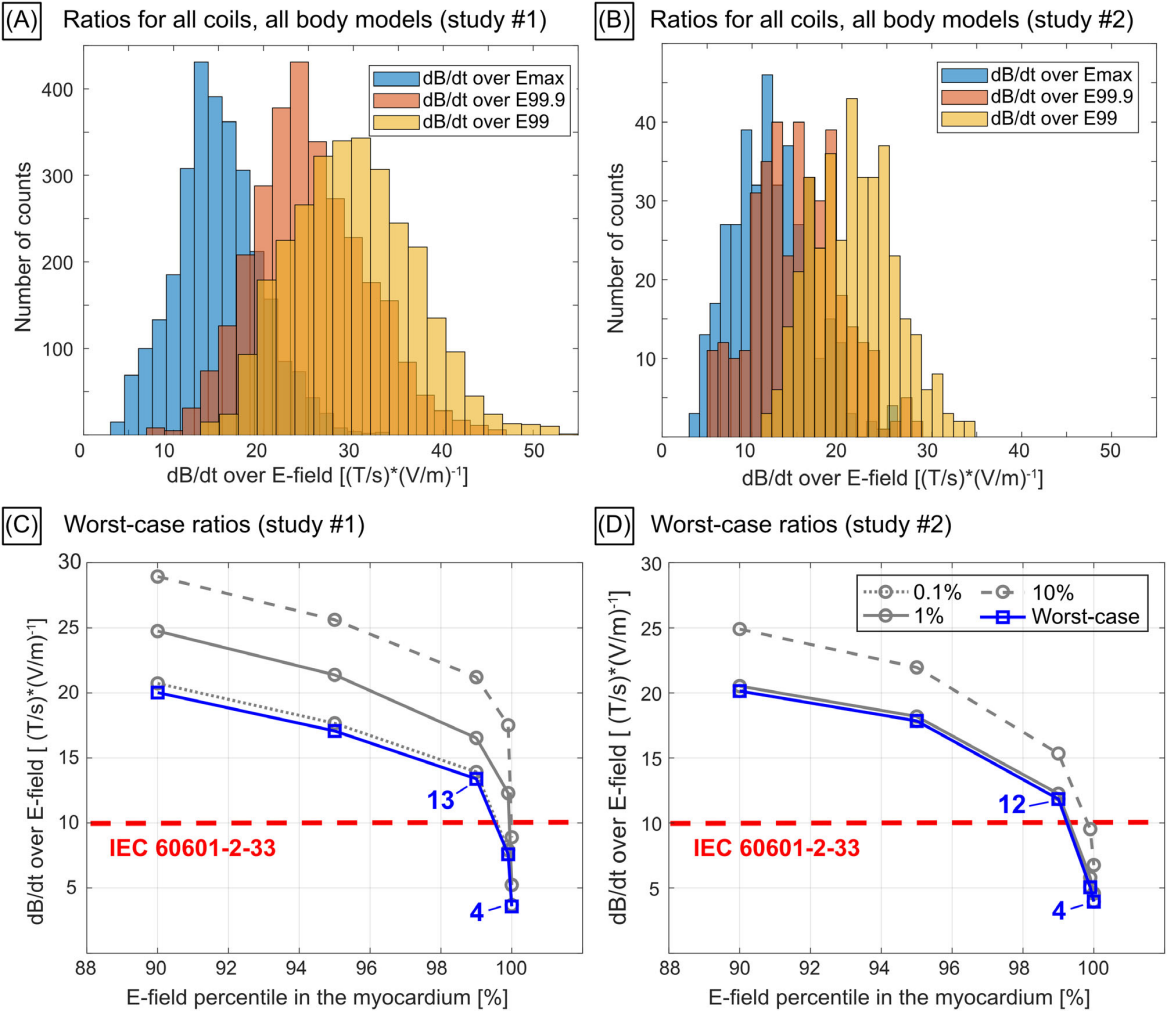
dB/dt‐over‐E‐field ratios evaluated using different E‐field thresholding values in the myocardial volume (Emax, E99.9, E99). (A) Histograms of dB/dt‐over‐E‐field ratios across all body models, all gradient systems, and axis combinations simulated in study #1. (B) Same plot for study #2. (C) Worst‐case dB/dt‐over‐E‐field ratios (blue curve) for varying E‐field thresholding in the myocardium (*x*‐axis) and 0.1st, 1st, and 10th percentiles of the dB/dt‐over‐E‐field distribution (gray curves) from study #1. (D) Same plot for study #2. The values on panels (C) and (D) refer to worst‐case dB/dt‐over‐Emax and dB/dt‐over‐E99 ratios, respectively.

Figure 5A,B show histograms of the dB/dt‐over‐E‐field ratios evaluated in studies #1 and #2 for the different GCs, body models, and axis combinations. For each study, we plot the resulting distribution without thresholding the individual myocardial E‐field maps (dB/dt‐over‐Emax) and with thresholds of 99.9% (dB/dt‐over‐E99.9) and 99% (dB/dt‐over‐E99). Figure S3 shows the same data for individual gradient systems. dB/dt‐over‐E99 ratios were more broadly distributed in study #1 (13–53 (T/s)*(V/m)^−1^) than in study #2 (12–35 (T/s)*(V/m)^−1^). Figure 5C,D show the worst‐case dB/dt‐over‐E‐field ratios (smallest ratio across all body model/GC combinations), as well as the 0.1st, 1st, and 10th percentile value of those ratios (gray curves). An important finding is that worst‐case ratios were similar in studies #1 and #2, that is, dB/dt‐over‐E99 = 13 (T/s)*(V/m)^−1^ (dB/dt‐over‐Emax = 4 (T/s)*(V/m)^−1^) in study #1 and dB/dt‐over‐E99 = 12 (T/s)*(V/m)^−1^ (dB/dt‐over‐Emax = 4 (T/s)*(V/m)^−1^) in study #2. The dB/dt‐over‐Emax, dB/dt‐over‐E99.9, and dB/dt‐over‐E99 ratios computed in both studies are openly available on GitHub at https://github.com/valerieklein/MRM_2025_dBdt_over_Efield_ratios.git.

## 4 Discussion

In this work, we performed EM field simulations in 75 detailed, heterogeneous body models and 18 realistic gradient systems to determine dB/dt‐over‐E‐field ratios in the heart expected during MRI. We performed two independent simulation studies with different coils, body models, E‐field simulation approaches, and post‐processing (study #1 was performed by VK, while study #2 was performed by JE). Study #1 simulated a larger range of body model/GC combinations (18 gradient systems × 4 axis combinations × 56 body models = 4032 simulations) than study #2 (5 gradient systems×4 axis combinations × 19 body models = 380 simulations). Study #1 found a broader distribution of ratios (dB/dt‐over‐E99 = 13–53 (T/s)*(V/m)^−1^) than study #2 (dB/dt‐over‐E99 = 12–35 (T/s)*(V/m)^−1^). We found that dB/dt‐over‐E99 ratios varied more across body models than across the investigated gradient systems (Figure S3). However, we found no correlation between dB/dt‐over‐E99 ratio and anatomical features of the body models such as weight, height, or BMI. We also found no correlation between dB/dt‐over‐E99 ratio and coil diameter or length. Note that the dB/dt‐over‐E99 ratio of gradient coil designs that differ greatly from the ones investigated in this work may be different.

Both studies found similar worst‐case dB/dt‐over‐E‐field ratios (study #1: dB/dt‐over‐E99 = 13 (T/s)*(V/m)^−1^ and study #2: dB/dt‐over‐E99 = 12 (T/s)*(V/m)^−1^). There are, however, also some differences between both studies. Specifically, the E‐fields simulated in study #2 are ~40% higher than in study #1 (Figure 4). To shed light on this discrepancy, we simulated one of the body models (Duke Virtual Family Project model with an enlarged heart) and one of the coils from study #2 (GC15, *Y*‐axis) using the EM pipeline of study #1 (Figure S4). We found that using the same mesh resolution as in study #2 (5 mm isotropic), both EM solvers predicted the same worst‐case landmark location and E99 value (difference = 3%). In other words, the EM solvers of study #1 and study #2 yield similar results when using the same body model, coil, and mesh resolution. An interesting finding of this comparison is that using an enlarged heart instead of the normal heart of the Duke body model increased E‐field values by 22%. This indicates that patients with cardiomegaly may be at a slightly higher risk of CS than the normal population, a fact that should probably be reflected in the final CS limit. Finally, to further probe the discrepancy between the results of studies #1 and #2 we investigated the effect of mesh resolution on E‐fields and showed that E‐field metrics stabilize for resolutions higher than or equal to 1 mm (Figures S5–S8). In particular, at 5 mm resolution (as used in study #2), E99 is 33% higher than at 1 mm resolution (as used in study #1). The combined effects of lower resolution and enlarged heart anatomy in study #2 may explain why the E‐fields simulated in study #2 are ~40% higher than those simulated in study #1.

Staircasing is a common and unavoidable artifact in E‐field simulations using hexahedral voxel models that can result in overestimation of the induced E‐fields at boundaries between tissues with different electrical conductivity [18, 19], such as the myocardium (*σ* = 0.385 S/m [15]), the lungs (*σ* = 0.101 S/m [15]) and blood pool (*σ* = 0.662 S/m [15]). A previous study [19] as well as our own simulations (Figures S5–S8) show that high‐resolution hexahedral meshes can have similar or even higher peak E‐field (Emax) values than low‐resolution meshes, and that staircasing does not decrease with increasing mesh resolution [19, 20]. We compared two different approaches to handle staircasing: (1) Thresholding of the E‐field maps at different percentile values, and (2) erosion of the outer voxel layer of the heart mask. Erosion of the outer voxel layer removes a substantial amount of data, depending on resolution (12% of all voxels at 0.5 mm, 23% at 1 mm), as can be seen in the E‐field maps (Figures S5 and S6) as well as in the E‐field histograms (Figure S7). E99 thresholding removes only 1% of all voxels regardless of mesh resolution. 99th percentile thresholding thus preserves more data than erosion of the myocardium at 1 mm mesh resolution. Both E99 and E95 values in the myocardium showed little variability across mesh resolutions compared to Emax (Figure S8). The 99th percentile thresholding is in line with safety guidelines issued by ICNIRP, which recommends that “for a specific tissue, the 99th percentile value of the electric field is the relevant value” when restricting adverse effects of induced E‐fields on excitable tissues [21], and has been supported by previous studies [19, 20].

Both studies #1 and #2 simulated E‐fields at different landmark locations by shifting the body models along the z‐direction to capture the worst‐case configuration for each gradient system, gradient axis, and body model. We found that displacements as small as ±5 cm along the z‐direction affected the myocardial E‐field by up to 70%. In study #1, we also assessed the impact of small body model shifts along the x‐ and y‐direction on the myocardial E‐fields in 6 body models and one gradient system. We found moderate myocardial E‐field changes of up to 8% for ±5 cm displacements along the x‐ and y‐directions, suggesting that those effects are not likely to affect the conclusion of this study.

We simulated all body models in head‐first supine position relative to the bore. However, clinical systems (including those simulated in this work) have a high degree of symmetry. We therefore expect our results to hold for all common body positions. The first symmetry is with respect to the isocenter z‐plane, which implies that both head‐first and feet‐first supine positions are accounted for in our simulations (this holds because we carefully determined the worst‐case landmark position for all coils and body models, which guarantees that there is no head‐first or feet‐first landmark position with a lower dB/dt‐over‐E‐field ratio than the ones plotted in Figure 5). The second coil symmetry is a 90° rotational symmetry around the z‐direction (parallel to B0) implying, to first order, that our simulations also reflect prone and lateral positions. This is not strictly correct because body shapes are different in the prone, lateral, and supine positions, but the impact of this effect on the myocardial E‐field is difficult to assess due to the scarcity of body models in prone and lateral positions.

In this work, we focused on whole‐body GCs and did not simulate special‐purpose systems such as head‐only gradients. Therefore, our conclusions only apply to whole‐body systems, which is in line with the current version of IEC 60601‐2‐33 [6]. Another limitation is that we only considered non‐pregnant adults. Pregnant women have a larger abdomen cross section than non‐pregnant patients, which could result in larger E‐fields in the myocardium and therefore lower dB/dt‐over‐E‐field ratios. This patient population should therefore be studied separately. We also did not model the many myocardial abnormalities encountered in patients. However, we point out that study #2 used enlarged heart models derived from MRI of heart failure patients to capture worst‐case myocardial E‐fields. Indeed, larger heart volumes likely result in higher myocardial E‐fields, and thus more conservative estimates of the dB/dt‐over‐E‐field ratio. Finally, we only evaluated the E‐field inside the myocardium to compute dB/dt‐over‐E‐field ratios as defined by IEC 60601‐2‐33 [6]. However, cardiac function can not only be affected by stimulation of the myocardium itself, but also by stimulation of the vagus nerve, which can modulate the heart rate [22]. Future investigations should compute E‐fields along the vagus nerve to ensure they are sub‐threshold at the level of the IEC cardiac stimulation limit. Doing so will require the use of body models that have an accurate description of the vagus nerve trajectory, such as the Zygote body models (American Fork, UT) that we previously used for PNS simulations [23, 24, 25, 26].

The dB/dt‐over‐E‐field ratio in IEC 60601‐2‐33 (10 (T/s)*(V/m)^−1^) likely reflects the worst case situation to be found in any human. Our simulations allow us to estimate the probability of dB/dt‐over‐E‐field ratios across the population and show that the lower tail of the distribution of dB/dt‐over‐E99 slightly exceeds this value. Considering the 10 ppb (10^−8^) acceptability threshold for a CS adverse event results in an E‐field rheobase of E_rheo_ = 2 V/m [6] in IEC 60601‐2‐33. We have re‐calculated this probability by computing the probability density distribution of a CS adverse event in units of dB/dt as a function of the choice of the specific dB/dt‐over‐E99 ratio used in the guideline (Figure S9). We only performed this calculation based on the data provided by study #1, as the E‐fields simulated in study #2 are artificially high due to the coarse mesh resolution (5 mm), which likely skewed the dB/dt‐over‐E99 ratios predicted by study #2 toward small values. We found that using the worst‐case simulated ratio for study #1 (13 (T/s)*(V/m)^−1^) leads to a vanishingly small probability (< 10^−15^). Our results indicate that a 10‐ppb likelihood (as used in IEC 60601‐2‐33) in a realistic population is reached at higher dB/dt‐over‐E99 ratios (30 (T/s)*(V/m)^−1^).

## 5 Conclusion

We performed two independent simulation studies of dB/dt‐over‐E‐field ratios for cardiac safety in MRI using realistic body models and gradient systems covering a large proportion of clinical scenarios. The two studies predicted different distributions of the dB/dt‐over‐E‐field ratios across the simulated body model/gradient coil combinations but found a similar worst‐case value of dB/dt‐over‐E99 of 13 (T/s)*(V/m)^−1^ (study #1) and 12 (T/s)*(V/m)^−1^ (study #2), respectively. The estimated probability of a CS adverse event remains well below the limit of 10 ppb currently used in IEC 60601‐2‐33 for the lowest predicted dB/dt‐over‐E99 ratios. Overall, our findings indicate that an increase of the dB/dt‐over‐E‐field ratio above 10 (T/s)*(V/m)^−1^ may be justified, which would allow for a more liberal use of gradient performance, thus ultimately enabling faster imaging and high MRI resolutions.

## Conflicts of Interest

Jonathan Edmonson is an employee of Medtronic. Matthias Gebhardt and Dominik Rattenbacher are employees of Siemens Healthineers. Johan S. van den Brink is an employee of Philips Healthcare. Michael Steckner is an employee of MKS Consulting. These companies had no role in study design, data collection and analysis, decision to publish, or preparation of the manuscript. All other authors declare no competing interests.

## Supporting information


**Figure S1:** (A) Height, weight, and BMI of the 56 adult XCAT body models (33 male, 23 female) used in simulation study #1. (B) Height, weight, and BMI of the 19 adult body models used in simulation study #2. (C) Body models' height and weight plotted onto the CAESAR data, which comprises data from 5000 adults from North America and Europe. The simulated body models cover a large range of the height and weight distribution of a representative human population. (D) Coil lengths and inner diameters (not bore size) of the X‐, Y‐, and Z‐gradient coils for each of the 18 simulated gradient systems. Exemplary gradient coils for which results are shown in Figures 1, 2, 3, and S2 are labeled.
**Figure S2:** Maximum intensity projections (MIPs) and slices of the E‐field distributions in the myocardium of different body models and GCs simulated in study #1. E‐field thresholding at different percentile values helps to reduce E‐field hotspots caused by staircasing artifacts.
**Figure S3:** Distributions of dB/dt‐over‐E‐field ratios evaluated in all models and for each gradient system (GC1‐GC18) for varying E‐field percentiles (dB/dt‐over‐Emax, dB/dt‐over‐E99.9, dB/dt‐over‐E99).
**Figure S4:** E‐field percentile metrics E95, E99, E99.9, and Emax (peak E‐field) at the “worst‐case” landmark location simulated in the Duke Virtual Family population body model with an enlarged heart loaded in GC15 (*Y*‐axis). E‐fields were simulated at 5 mm isotropic resolution using the EM solvers of study #1 (blue bars) and study #2 (red bars). E99 differs by only 3% between both studies, indicating that the EM solvers of both studies yield similar results for the same body model, coil, and resolution.
**Figure S5:** Maximum intensity projections of the E‐field in the myocardium of a male body model (Zygote, American Fork, UT) loaded in GC10 (*X*‐axis). The E‐field was simulated at hexahedral mesh resolutions between 0.5 mm and 5 mm (rows). The columns (left to right) show full E‐field maps, E‐fields thresholded by 99.9th, 99th, and 95th percentile values, and E‐field maps after erosion of the outer voxel layer of the heart.
**Figure S6:** Coronal slices of the E‐field in the myocardium of a male body model (Zygote, American Fork, UT) loaded in GC10 (*X*‐axis). The E‐field was simulated at hexahedral mesh resolutions between 0.5 mm and 5 mm (rows). The columns (left to right) show full E‐field maps, E‐fields thresholded by 99.9th, 99th, and 95th percentile values, and E‐field maps after erosion of the outer voxel layer of the heart.
**Figure S7:** Histograms of the E‐field in all voxels of the myocardium of a male body model (Zygote, American Fork, UT) loaded in GC10 (*X*‐axis). The E‐field was simulated at hexahedral mesh resolutions between 0.5 mm and 5 mm (rows). The columns (left to right) show histograms for full E‐field maps, E‐fields thresholded by 99.9th, 99th, and 95th percentile values, and E‐field maps after erosion of the outer voxel layer of the heart.
**Figure S8:** Maximum E‐field (Emax), 99.9th percentile E‐field (E99.9), 99th percentile E‐field (E99), and 95th percentile E‐field (E95) in the heart of a male body model (Zygote, American Fork, UT) loaded in GC10 (*X*‐axis) as a function of hexahedral mesh resolution.
**Figure S9:** Probability of a CS adverse event for different dB/dt‐over‐E99 ratios computed in study #1. The worst‐case dB/dt‐over‐E99 ratio of 13 (T/s)*(V/m)^−1^ leads to a probability of < 10^−15^.

## Data Availability

The data that support the findings of this study are openly available on GitHub at https://github.com/valerieklein/MRM_2025_dBdt_over_Efield_ratios.git.
