# Supplementary material for: Simulation of dB/dt‐Over‐Electric Field Cardiac Magnetostimulation Safety Ratios in 75 Body Models and 18 Gradient Systems
Source: Magn Reson Med. 2025 Oct 7;95(3):1753–61. doi: 10.1002/mrm.70130 (PMC12746381; doi:10.1002/mrm.70130)
Supplement: Supplementary file 1 — Figure S1: (A) Height, weight, and BMI of the 56 adult XCAT body models (33 male, 23 female) used in simulation study #1. (B) Height, weight, and BMI of the 19 adult body models used in simulation study #2. (C) Body models' height and weight plotted onto the CAESAR data, which comprises data from 5000 adults from North America and Europe. The simulated body models cover a large range of the height and weight distribution of a representative human population. (D) Coil lengths and inner diameters (not bore size) of the X‐, Y‐, and Z‐gradient coils for each of the 18 simulated gradient systems. Exemplary gradient coils for which results are shown in Figures 1, 2, 3, and S2 are labeled. Figure S2: Maximum intensity projections (MIPs) and slices of the E‐field distributions in the myocardium of different body models and GCs simulated in study #1. E‐field thresholding at different percentile values helps to reduce E‐field hotspots caused by staircasing artifacts. Figure S3: Distributions of dB/dt‐over‐E‐field ratios evaluated in all models and for each gradient system (GC1‐GC18) for varying E‐field percentiles (dB/dt‐over‐Emax, dB/dt‐over‐E99.9, dB/dt‐over‐E99). Figure S4: E‐field percentile metrics E95, E99, E99.9, and Emax (peak E‐field) at the “worst‐case” landmark location simulated in the Duke Virtual Family population body model with an enlarged heart loaded in GC15 (Y‐axis). E‐fields were simulated at 5 mm isotropic resolution using the EM solvers of study #1 (blue bars) and study #2 (red bars). E99 differs by only 3% between both studies, indicating that the EM solvers of both studies yield similar results for the same body model, coil, and resolution. Figure S5: Maximum intensity projections of the E‐field in the myocardium of a male body model (Zygote, American Fork, UT) loaded in GC10 (X‐axis). The E‐field was simulated at hexahedral mesh resolutions between 0.5 mm and 5 mm (rows). The columns (left to right) show full E‐field maps, E‐fields thresholded by 99.9 [file MRM-95-1753-s001.docx]

**Simulation of dB/dt-over-electric field cardiac magnetostimulation safety ratios in 75 body models and 18 gradient systems**

Valerie Klein^1,2*^, Jonathan Edmonson^3*^, Mathias Davids^1^, Natalie G. Ferris^1,4^, Matthias Gebhardt^5^, Dominik Rattenbacher^5^, Johan S. van den Brink^6^, Michael Steckner^7^, Lawrence L. Wald^1,2,4^, Bastien Guérin^1,2^

^1^A. A. Martinos Center for Biomedical Imaging, Department of Radiology, Massachusetts General Hospital, Charlestown, MA, United States;

^2^Harvard Medical School, Boston, MA, United States;

^3^Medtronic Cardiac Rhythm Management, Minneapolis, MN, United States;

^4^Harvard-MIT Division of Health Sciences and Technology, Cambridge, MA, United States;

^5^Siemens Healthineers, Erlangen, Germany;

^6^Philips Healthcare, Best, The Netherlands;

^7^MKS Consulting, Beachwood, OH, United States

*These authors contributed equally to this work.

**Supporting Information Figures**


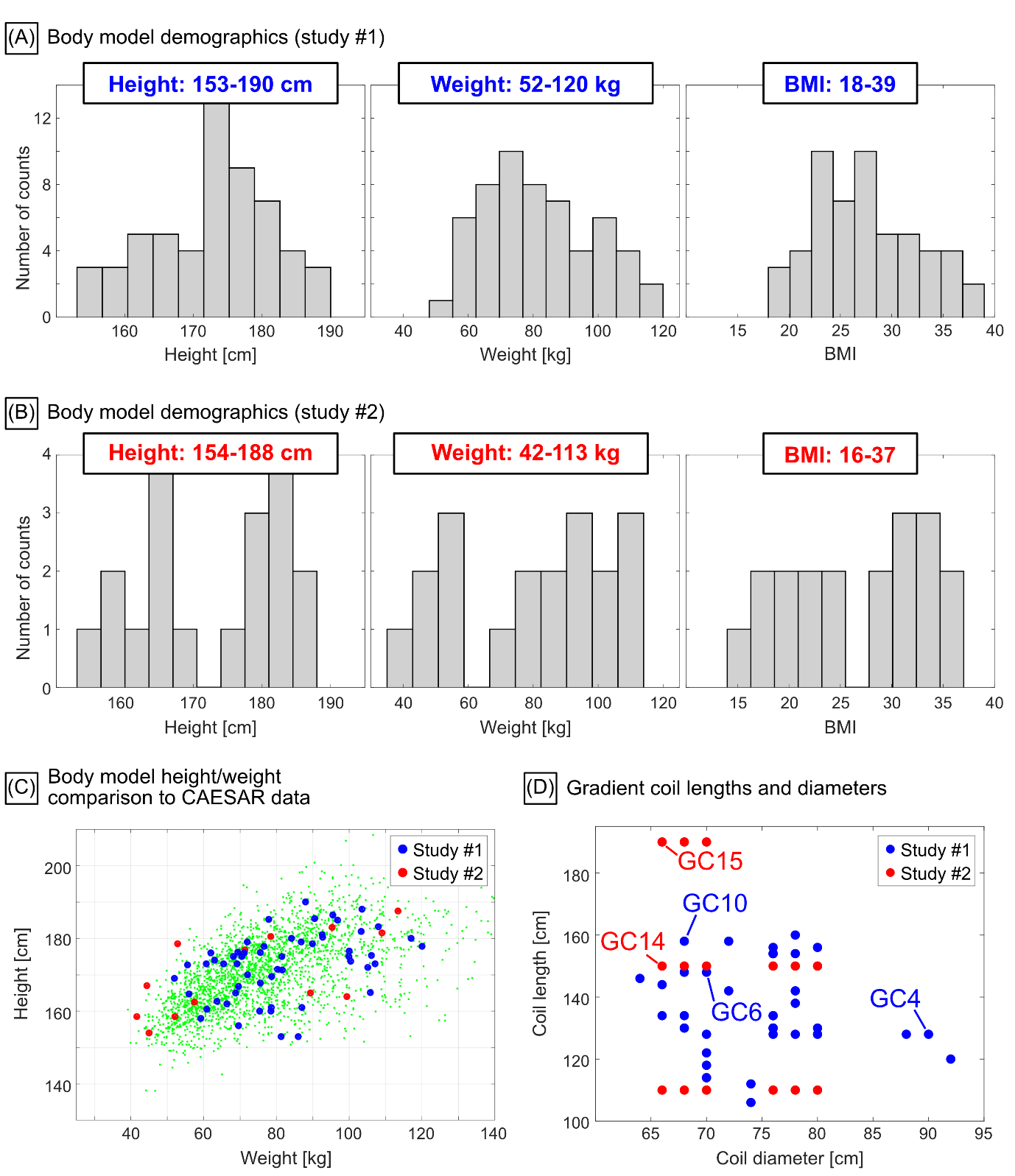


**Supporting Information Figure S1: A)** Height, weight, and BMI of the 56 adult XCAT body models (33 male, 23 female) used in simulation study #1. **B)** Height, weight, and BMI of the 19 adult body models used in simulation study #2.**C)** Body models’ height and weight plotted onto the CAESAR data, which comprises data from 5,000 adults from North America and Europe. The simulated body models cover a large range of the height and weight distribution of a representative human population. **D)** Coil lengths and inner diameters (not bore size) of the X-, Y-, and Z-gradient coils for each of the 18 simulated gradient systems. Exemplary gradient coils for which results are shown in Figures 1, 2, 3, and S2 are labeled.


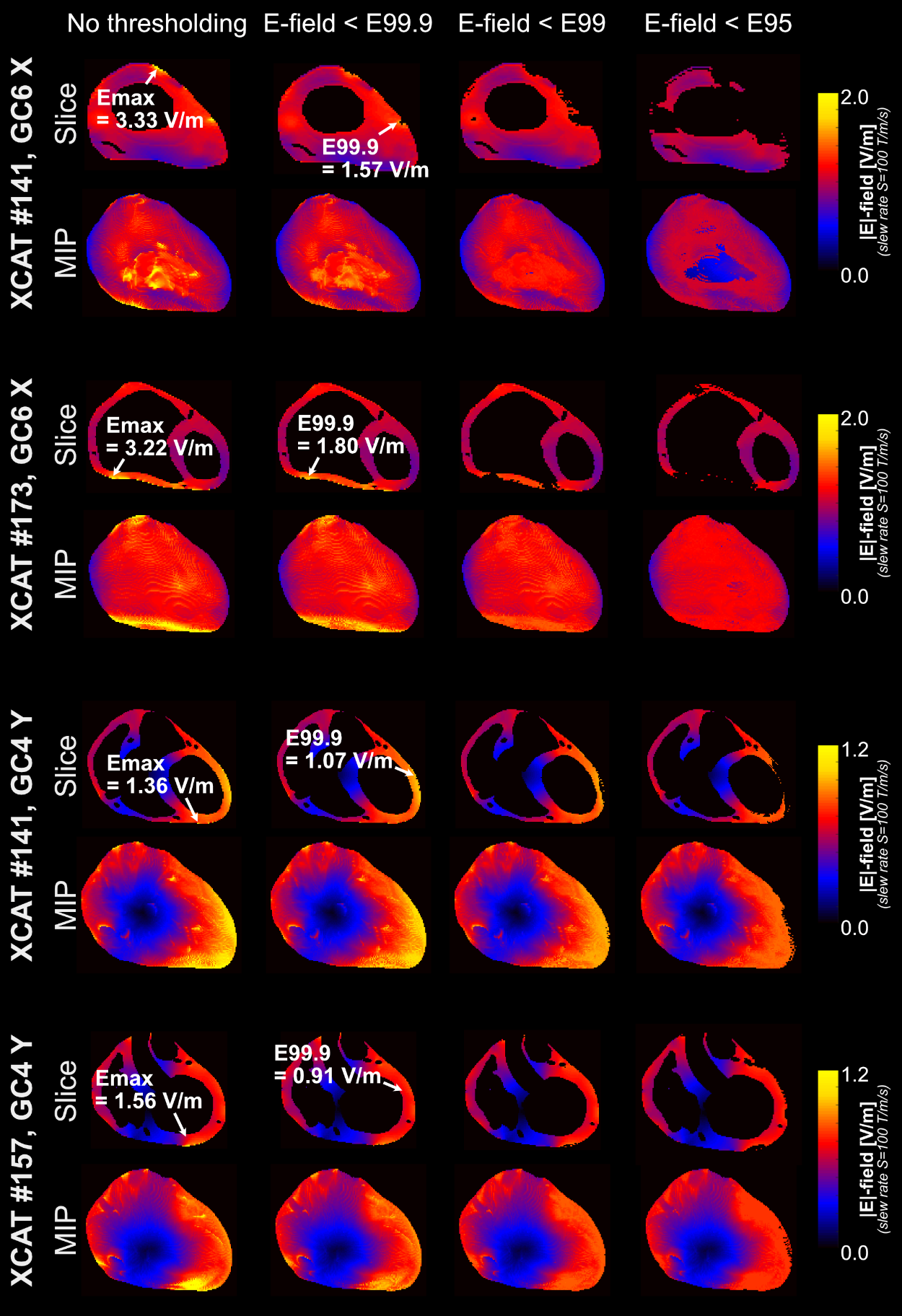


**Supporting Information Figure S2:** Maximum intensity projections (MIPs) and slices of the E-field distributions in the myocardium of different body models and GCs simulated in study #1. E-field thresholding at different percentile values helps to reduce E-field hotspots caused by staircasing artifacts.


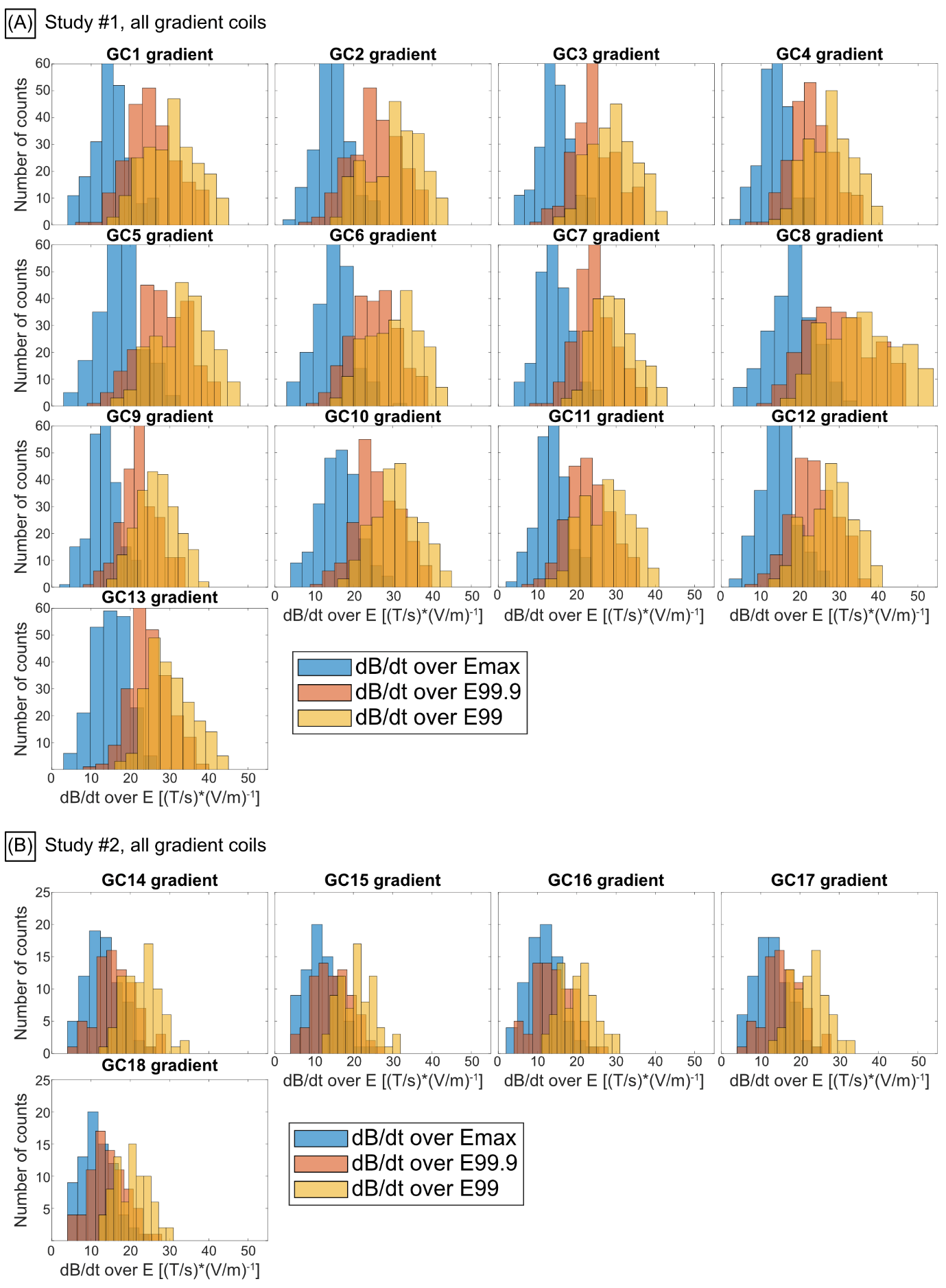


**Supporting Information Figure S3:** Distributions of dB/dt-over-E-field ratios evaluated in all models and for each gradient system (GC1-GC18) for varying E-field percentiles (dB/dt-over-Emax, dB/dt-over-E99.9, dB/dt-over-E99).


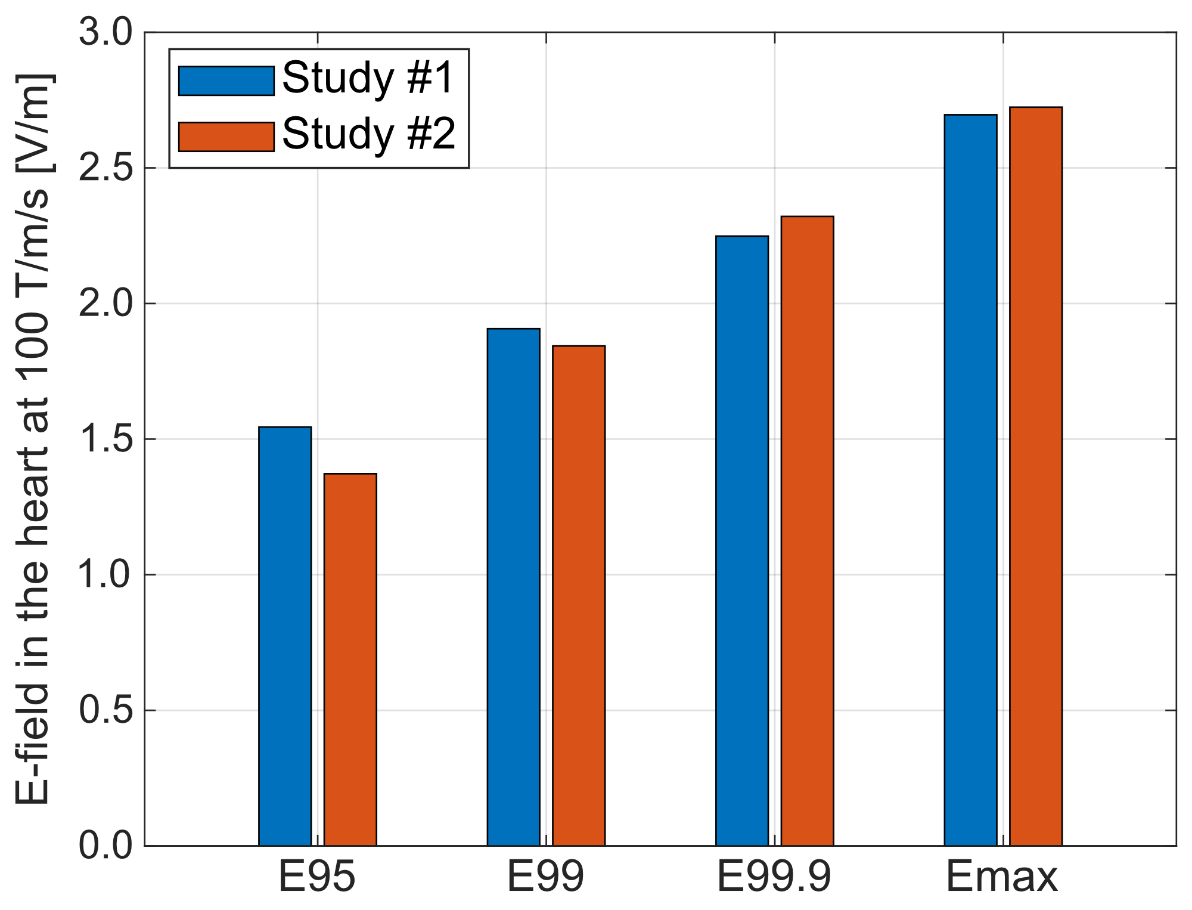


**Supporting Information Figure S4:** E-field percentile metrics E95, E99, E99.9, and Emax (peak E-field) at the “worst-case” landmark location simulated in the Duke Virtual Family population body model with an enlarged heart loaded in GC15 (Y-axis). E-fields were simulated at 5 mm isotropic resolution using the EM solvers of study #1 (blue bars) and study #2 (red bars). E99 differs by only 3% between both studies, indicating that the EM solvers of both studies yield similar results for the same body model, coil, and resolution.


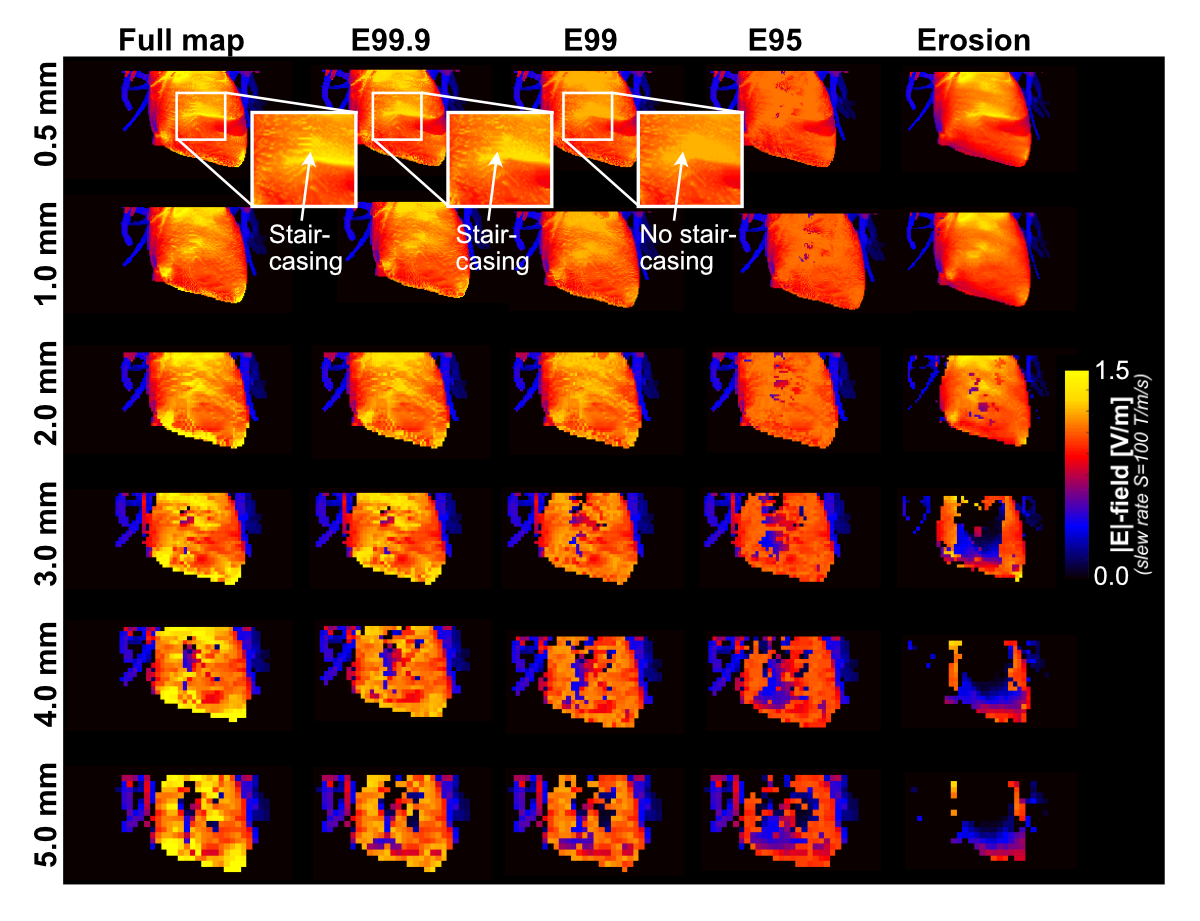


**Supporting Information Figure S5:** Maximum intensity projections of the E-field in the myocardium of a male body model (Zygote, American Fork, UT) loaded in GC10 (X-axis). The E-field was simulated at hexahedral mesh resolutions between 0.5 mm and 5 mm (rows). The columns (left to right) show full E-field maps, E-fields thresholded by 99.9^th^, 99^th^, and 95^th^ percentile values, and E-field maps after erosion of the outer voxel layer of the heart.


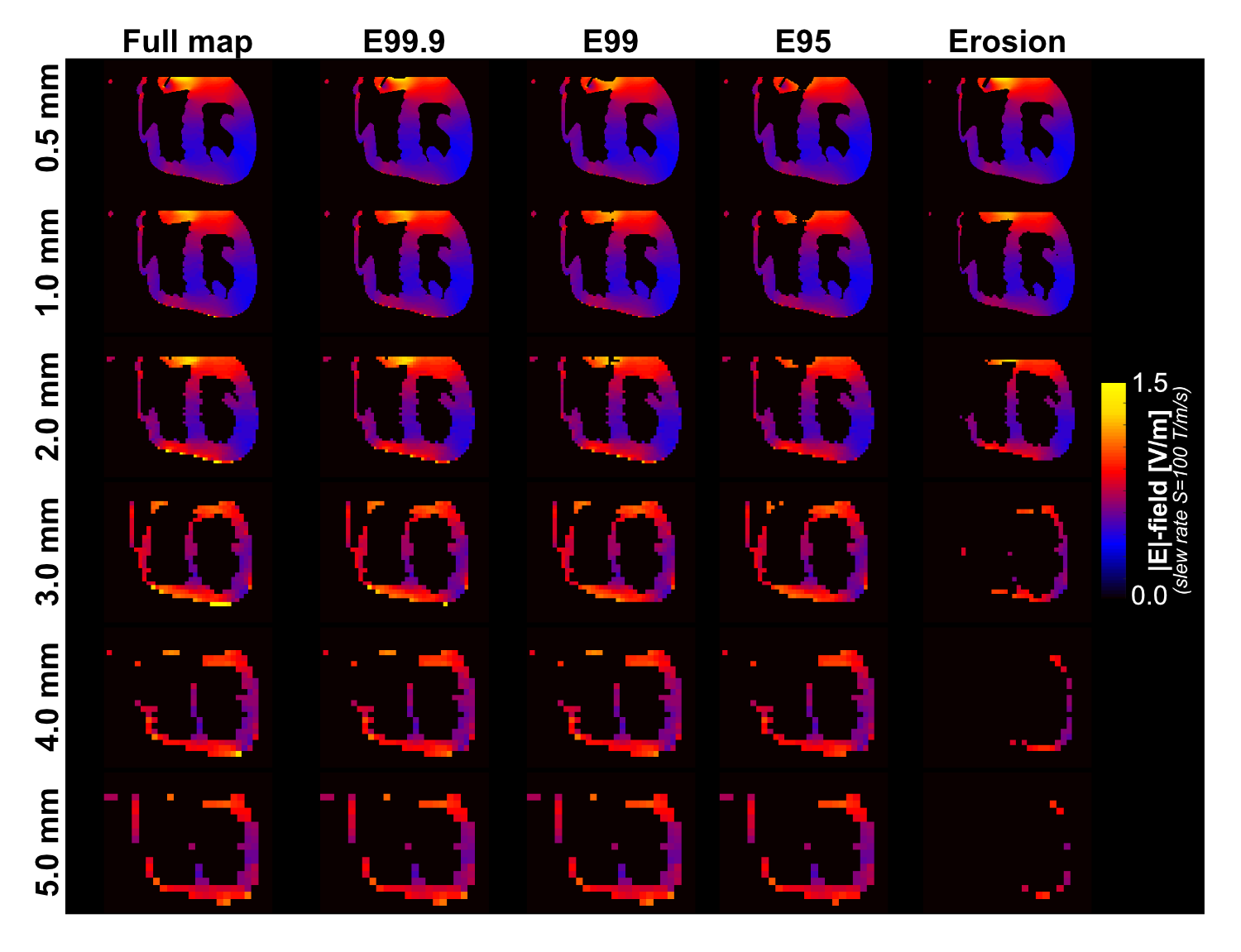


**Supporting Information Figure S6:** Coronal slices of the E-field in the myocardium of a male body model (Zygote, American Fork, UT) loaded in GC10 (X-axis). The E-field was simulated at hexahedral mesh resolutions between 0.5 mm and 5 mm (rows). The columns (left to right) show full E-field maps, E-fields thresholded by 99.9^th^, 99^th^, and 95^th^ percentile values, and E-field maps after erosion of the outer voxel layer of the heart.


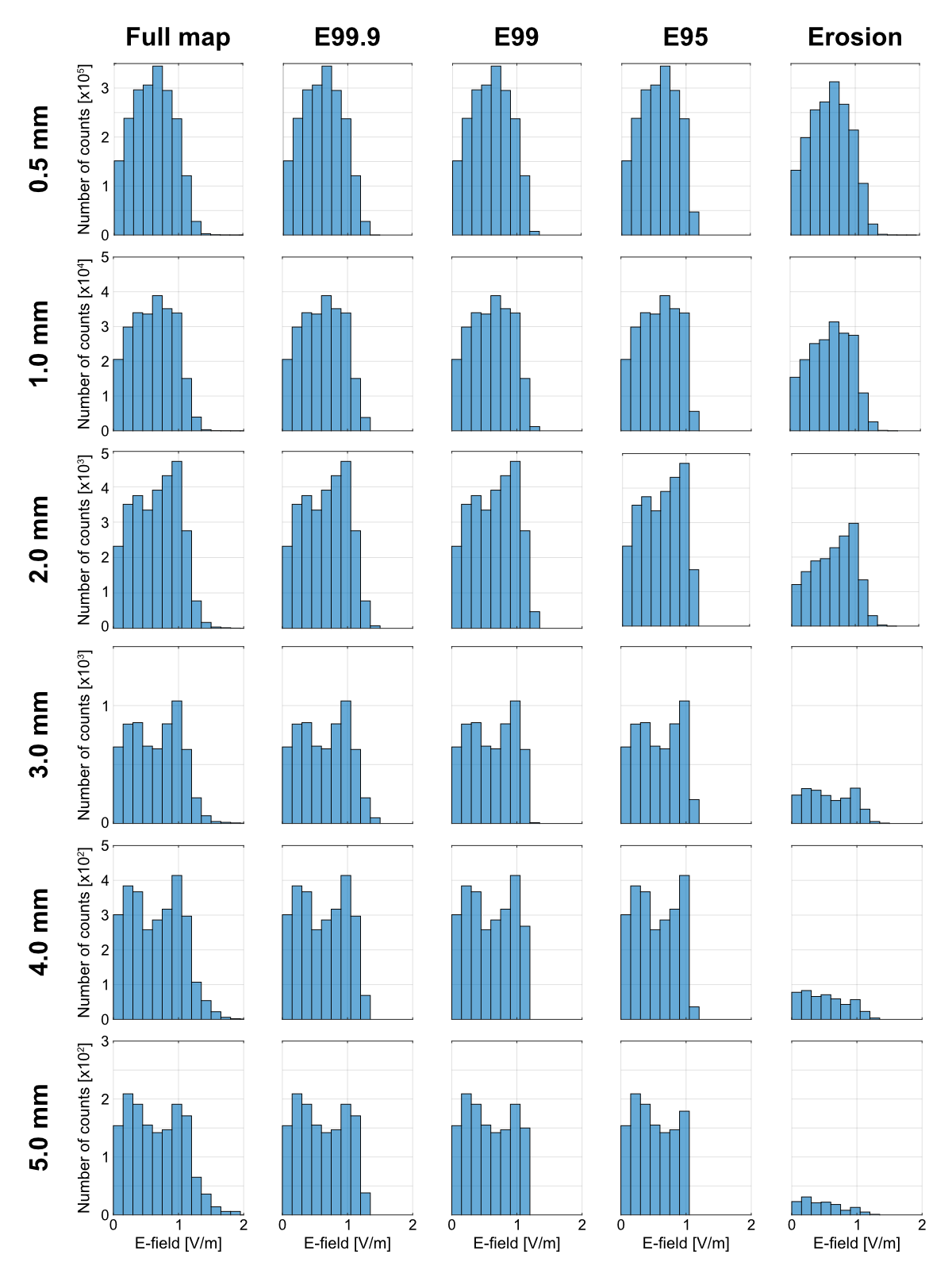


**Supporting Information Figure S7:** Histograms of the E-field in all voxels of the myocardium of a male body model (Zygote, American Fork, UT) loaded in GC10 (X-axis). The E-field was simulated at hexahedral mesh resolutions between 0.5 mm and 5 mm (rows). The columns (left to right) show histograms for full E-field maps, E-fields thresholded by 99.9^th^, 99^th^, and 95^th^ percentile values, and E-field maps after erosion of the outer voxel layer of the heart.


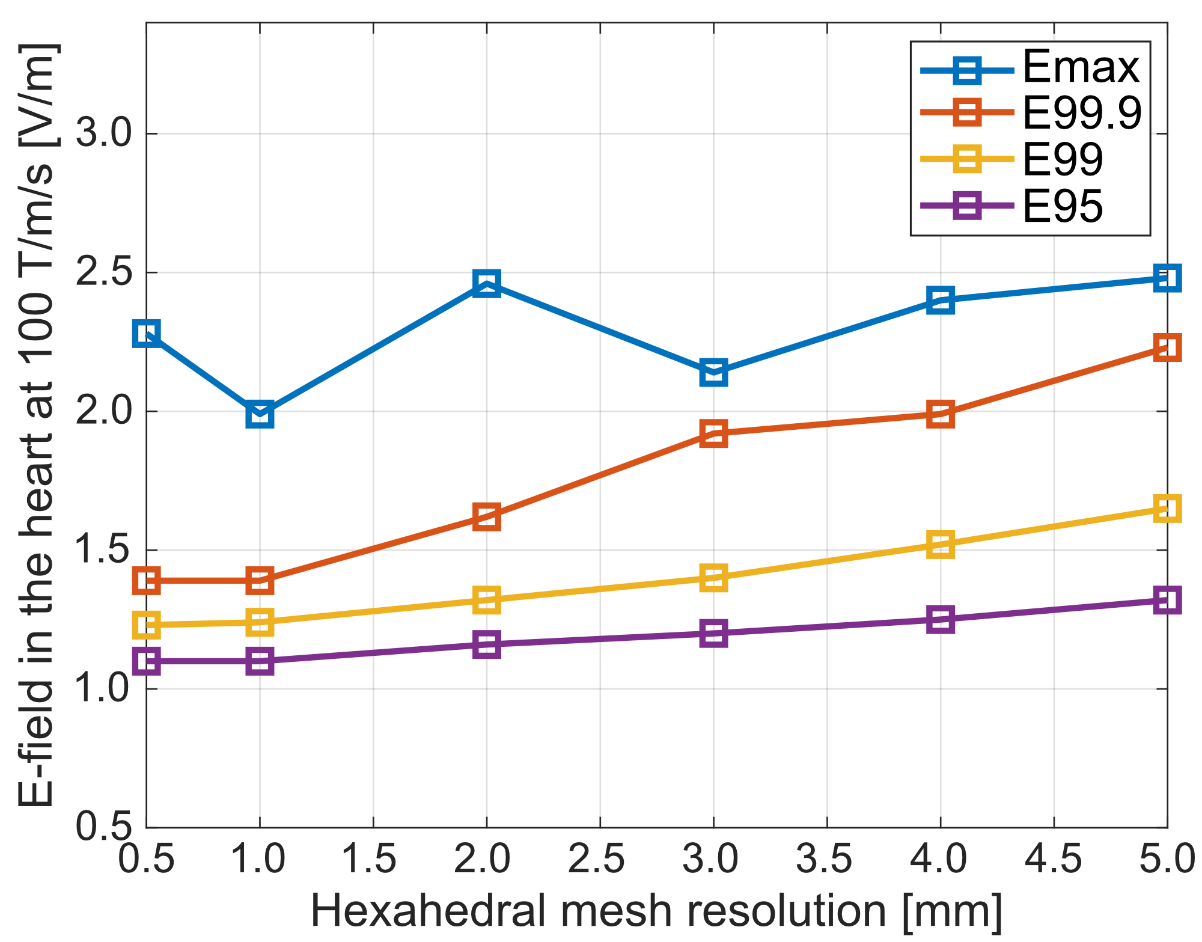


**Supporting Information Figure S8:** Maximum E-field (Emax), 99.9^th^ percentile E-field (E99.9), 99^th^ percentile E-field (E99), and 95^th^ percentile E-field (E95) in the heart of a male body model (Zygote, American Fork, UT) loaded in GC10 (X-axis) as a function of hexahedral mesh resolution.


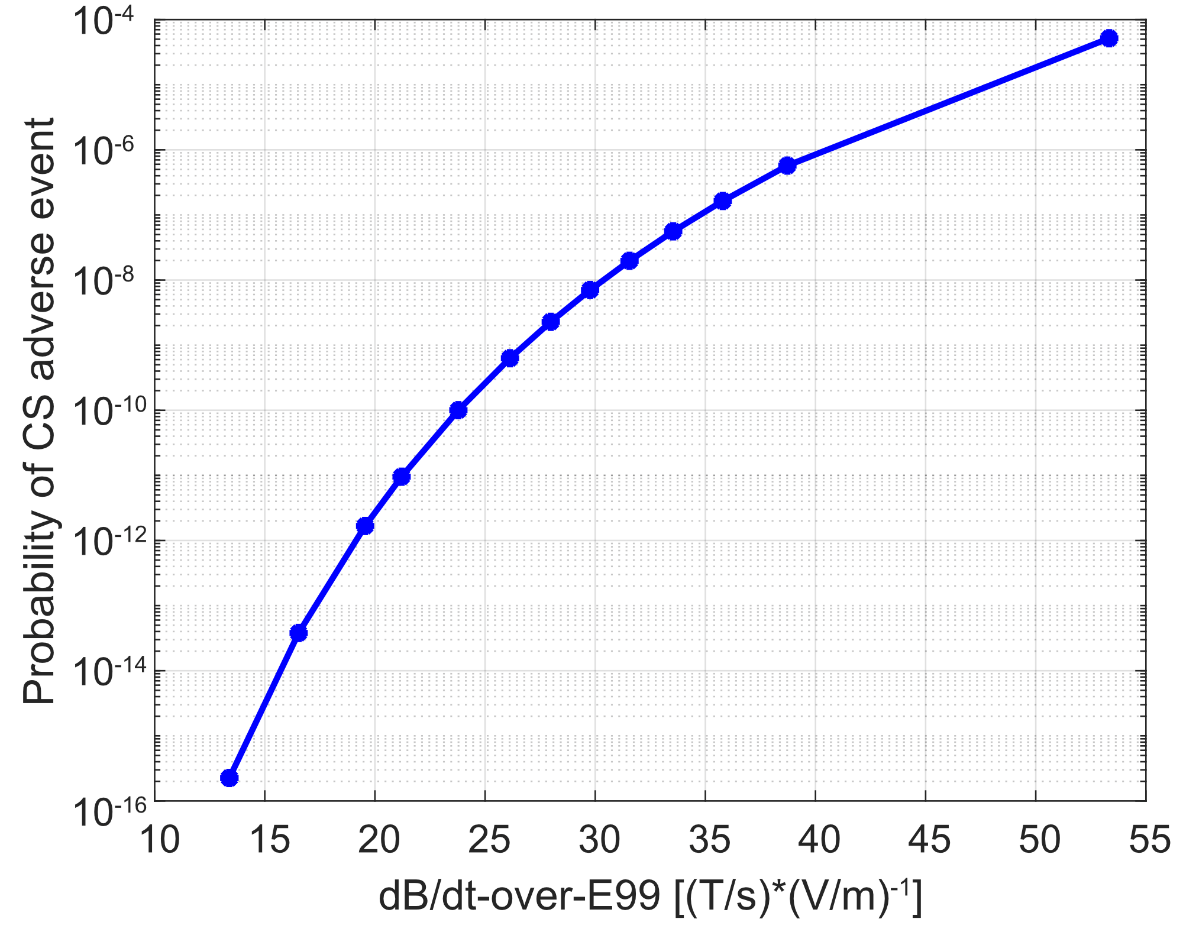


**Supporting Information Figure S9:** Probability of a CS adverse event for different dB/dt-over-E99 ratios computed in study #1. The worst-case dB/dt-over-E99 ratio of 13 (T/s)*(V/m)^-1^ leads to a probability of <10^-15^.
